# The association between peripheral blood inflammatory markers and anti-VEGF treatment response in patients with type 2 diabetic macular edema

**DOI:** 10.3389/fmed.2025.1653753

**Published:** 2025-10-16

**Authors:** Wenting Gu, Min Wang, Zhizhe Li, Tianqi Xu

**Affiliations:** ^1^Department of Ophthalmology, The Affiliated Suzhou Hospital of Nanjing Medical University, Suzhou Municipal Hospital, Suzhou, Jiangsu, China; ^2^Department of Ophthalmology, The Second Affiliated Hospital of Nantong University, Nantong, Jiangsu, China

**Keywords:** peripheral blood, leukocytes, systemic inflammation, diabetic macular edema, intravitreal anti-vascular endothelial growth factor

## Abstract

**Aim:**

This study aimed to investigate the correlation between serum inflammatory markers of patients with diabetic macular edema (DME) and the efficacy of intravitreal anti-vascular endothelial growth factor (VEGF).

**Methods:**

This was a single-center, prospective cohort study. Peripheral blood cell analysis was performed on 40 patients with confirmed type 2 diabetes complicated by DME, 40 healthy individuals, and 40 patients with confirmed type 2 diabetes without diabetic retinopathy. Neutrophil-to-lymphocyte ratio (NLR), platelet-to-lymphocyte ratio (PLR), monocyte-to-lymphocyte ratio (MLR), and systemic immune-inflammation index [SII; (neutrophil count × platelet count)/lymphocyte count] were calculated. All patients with DME received three monthly intravitreal injections of anti-VEGF agents (ranibizumab). Correlation analyzes and linear regression models were used to investigate the relationships between systemic inflammatory markers and best-corrected visual acuity (BCVA) and central macular thickness (CMT) before and after anti-VEGF treatment in DME patients.

**Results:**

The NLR, PLR, and SII values in the DME group differed significantly from those in both the healthy and non-diabetic retinopathy (NDR) groups. Significant differences in MLR values were observed between the healthy and NDR groups. After a 3-month follow-up (following three injections), BCVA and CMT showed significant improvement before and after anti-VEGF treatment in DME patients. However, there were no significant differences in NLR, PLR, and SII before and after anti-VEGF treatment. MLR was significantly different before and after treatment. BCVA in the DME group before anti-VEGF treatment was positively correlated with NLR, PLR, and SII. CMT before anti-VEGF treatment was positively correlated with NLR, PLR, MLR, and SII. NLR, PLR, MLR, and SII were significantly correlated with BCVA and CMT. In multivariate linear regression analysis, only NLR was significantly correlated with CMT.

**Conclusion:**

The efficacy of anti-VEGF in DME is correlated with serum inflammatory markers. Additionally, NLR, PLR, MLR, and SII may serve as potential markers for DME treatment decisions. The finding that NLR remained significant in the multivariate analysis highlights its potential value as a simple, accessible prognostic biomarker for stratifying patients who may respond suboptimally to anti-VEGF treatment.

## Introduction

Diabetic retinopathy (DR) is the most common ocular complication of diabetes (1) and the leading cause of visual impairment in working-age adults (2). Retinal microvascular leakage and obstruction caused by chronic progressive diabetes can lead to a series of fundal lesions, such as microhemangiomas, rigid exudation, cotton wool patches, neovascularization, vitreous proliferation, diabetic macular edema (DME), and even retinal detachment (3). Among these, DME is one of the most common causes of visual impairment; its global incidence is expected to increase by approximately 25% by 2030, reaching approximately 24 million cases (4).

Early diagnosis and treatment of DME are key to saving vision. Early detection of DME relies on optical coherence tomography (OCT) (5), which is non-invasive and highly repeatable. The current first-line treatment for DME is intravitreal anti-vascular endothelial growth factor (VEGF) treatment, which provides better visual acuity improvement than traditional panretinal photocoagulation (6). VEGF promotes vascular permeability, extracellular matrix degeneration, and vascular endothelial cell migration, proliferation, and angiogenesis (7). This increased vascular permeability allows proteins, lipids, and other plasma components to infiltrate retinal tissue, resulting in DME (8). VEGF is also an inflammatory mediator in DME, which stimulates the secretion of other cytokines and chemokines, amplifying the inflammatory response within the retinal tissue (9). Nevertheless, some patients still experience DME relapse despite receiving intravitreal injection of anti-VEGF drugs. Approximately 40% of patients have chronic persistent macular edema (10). As a biomarker of DME, VEGF is difficult to detect and cannot be evaluated in a timely manner. Therefore, identifying alternative biomarkers that are easily accessible and measurable is crucial for predicting and evaluating the efficacy of anti-VEGF therapy in DME. A stronger transition to the advantages of peripheral blood markers should be made.

Whether in animal models or diabetic patients, inflammation plays a significant role in the pathogenesis of DR. Chronic low-grade inflammation is widespread at various stages of DR (11). Generally, chronic inflammation in diabetes will trigger an inflammatory cell response in the body, which will then cause capillary dysfunction and eventually lead to DR. Studies have confirmed that leukocytosis is a key step in the early stage of DR. DR is associated with increased innate cellular immunity (especially neutrophils) and decreased adaptive cellular immunity (especially lymphocytes) (12). In recent years, serum inflammatory markers have been confirmed as biomarkers for the presence and development of DME (13). High SII values can predict the risk of early microvascular and macrovascular complications as well as mortality in patients with type 2 diabetic retinopathy (14). NLR, MPV, SII, and LMR were related to PDR, and incorporating them into the comprehensive risk prediction model could have practical value (15). However, the association between these inflammatory markers and the specific response of DME patients to anti-VEGF therapy currently lacks in-depth research, which is a critical knowledge gap that needs to be filled.

The purpose of this study is to evaluate the relationship between peripheral blood inflammation indices (NLR, PLR, MLR, and SII) and anti-VEGF treatment response in patients with type 2 DME.

## Materials and methods

**Figure 1:**
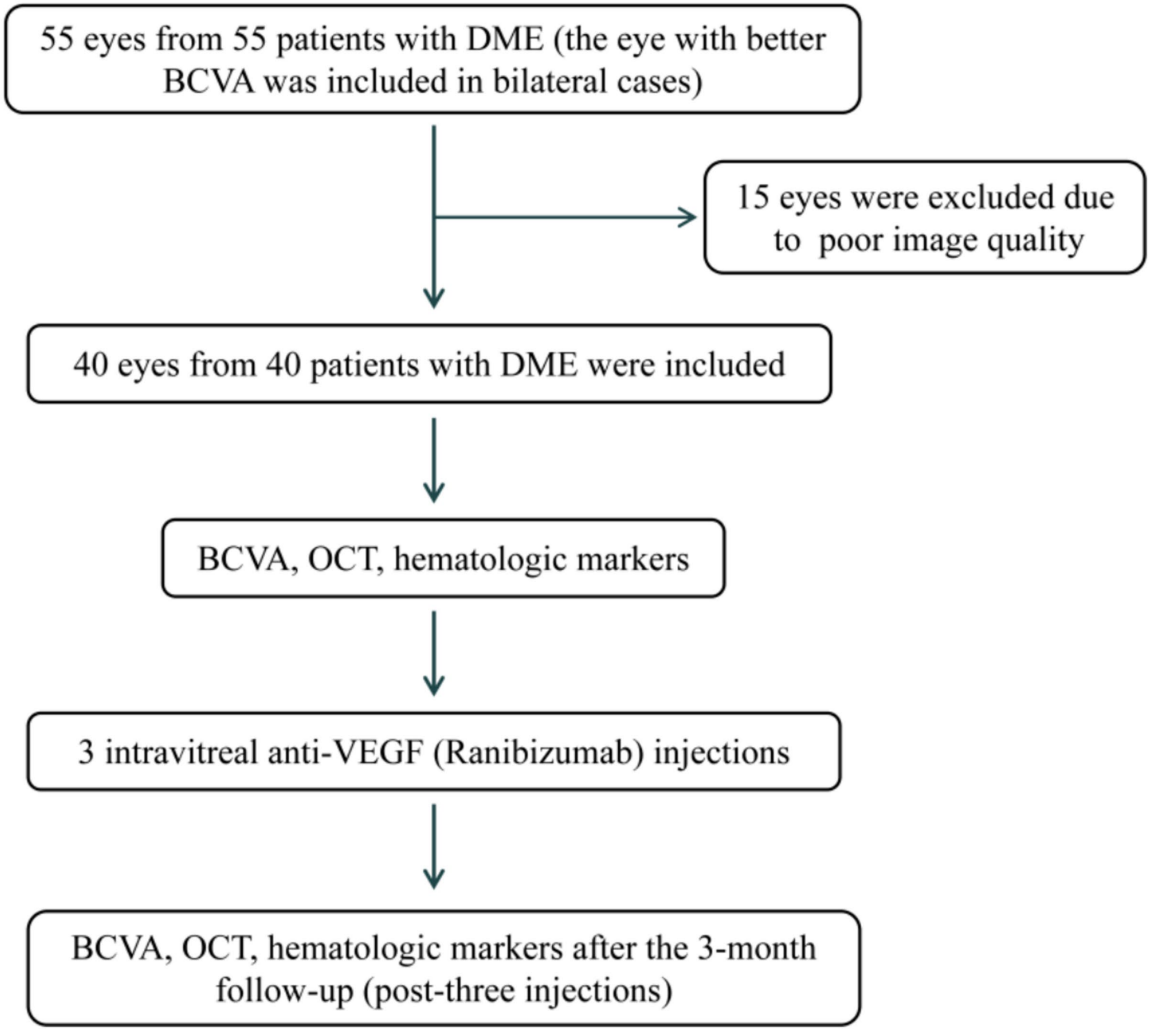
Correlation between BCVA and leucocyte ratios in the DME group before anti-VEGF treatment. DME, type 2 diabetes mellitus complicated with macular edema; BCVA, best-corrected visual acuity; OCT, optical coherence tomography.

In this observational cohort study, the observation group comprised 55 eyes of 55 patients diagnosed with type 2 diabetes mellitus complicated with macular edema (DME) from the Department of Ophthalmology, Nanjing Medical University Affiliated Suzhou Hospital, and Suzhou Municipal Hospital. Fifteen eyes were excluded because of poor image quality (Figure 1). All patients were required to receive intravitreal anti-VEGF (ranibizumab) injection once a month for at least 3 months. In the control group, we enrolled 40 healthy individuals who received physical examinations in the Department of Physical Examination of Nanjing Medical University Affiliated Suzhou Hospital, Suzhou Municipal Hospital, and 40 patients with type 2 diabetes without diabetic retinopathy diagnosed in the Department of Endocrinology. All participants were collected from July 2023 to June 2024.

The inclusion criteria were as follows: (1) patients aged ≥18 years, (2) patients who met the diagnostic criteria for type 2 diabetes mellitus combined with macular edema, and (3) patients who voluntarily enrolled in the study. Patients (1) whose pupils could not be dilated or whose dioptric interstitial space was cloudy so that the fundus could not be observed; (2) who had been treated with anti-VEGF, corticosteroids, or immunosuppressants within the previous 6 months; (3) with a previous history of other ocular diseases; and (4) with systemic diseases, such as cancer and diseases of the immune system affecting hematological indicators were excluded. Age, sex, the presence or absence of hypertension, basic parameters of body mass index (BMI), and glycosylated hemoglobin (HbA1c) were collected in all observation and control groups. Hematological indices included neutrophil, lymphocyte, monocyte, and platelet counts. Neutrophil-to-lymphocyte ratio (NLR), platelet-to-lymphocyte ratio (PLR), monocyte-to-lymphocyte ratio (MLR), and systemic immune-inflammation index [SII, (neutrophil count × platelet count)/lymphocyte count] were calculated. An automatic blood cell analyzer (BC-7500, Mindray, China) was used to measure a complete blood count. In the observation group, hematologic markers were collected before the first anti-VEGF treatment and 1 month after the third anti-VEGF treatment. SII, NLR, PLR, and MLR were measured according to previous studies (16–19). All blood samples were collected under fasting conditions.

All patients with type 2 diabetes and macular edema were required to undergo a complete eye examination before the first anti-VEGF treatment and 1 month after the third anti-VEGF treatment. The exam included best-corrected visual acuity (BCVA), slit lamp examination, fundus examination, OCT (OCT spectral domain, Zeiss-Humphrey, United States), and fluorescein fundus angiography (FFA). The BCVA evaluated the visual acuity changes based on the international standard visual acuity chart results, and the visual acuity was converted into the equivalent logMAR visual acuity chart value for statistical analysis. After mydriasis was achieved using 0.5% tropicamide for 30 min, the anterior segment and fundus were examined using a slit-lamp microscope and pre-set lens. After the patients in the observation group had fully dilated pupils, the foveae were scanned using a Fast Macular Thickness Map and a Macular Thickness Map on OCT. The scanning length was 6 mm. Images were analyzed using the Retinal Thickness method to measure the central macular thickness (CMT), which is the thickness of the retinal neuroepithelium. The average fovea thickness, within a 1 mm diameter, was used for comparison.

In this study, all enrolled patients with DME received intravitreal injection of ranibizumab (0.5 mg/0.05 mL) as the initial anti-VEGF treatment. All injections were performed by two associate chief physicians with over 5 years of specialized experience in retinal diseases. The procedure was strictly standardized: preoperative topical anesthesia was administered, and the conjunctival sac was disinfected with 5% povidone-iodine to prevent infection. The injection site was selected 3.5–4.0 mm posterior to the temporal corneal limbus, using a 30G needle for vertical insertion and slow injection. Postoperative assessments included checking the pulse of the central retinal artery and measuring intraocular pressure. The design and execution of this protocol were based on the American Academy of Ophthalmology (AAO) clinical guidelines for diabetic retinopathy to ensure treatment safety and consistency (20).

### Statistical methods

SPSS 27.0 version (IBM, New York, United States) was used for statistical analysis. The Shapiro–Wilk (S–W) test was used to determine the normality of continuous variables. Continuous variables conforming to a normal distribution are presented by mean±standard deviation. Univariate analysis of variance (ANOVA) was used to determine differences between groups, and Bonferroni was used for the adjustment of multiple testing. Non-normal distributed data are expressed as median (lower quartile to upper quartile); between-group differences were determined using a Mann–Whitney U-test or Kruskal–Wallis test, whereas intra-group data before and after treatment were compared using the Wilcoxon rank-sum matching test. Categorical variables are expressed as numerical values and percentages (%), and the chi-square test was used for data comparison. The Spearman correlation test (HbA1c was included as a covariate) was used to determine the correlation between baseline logMAR, CMT, and systemic inflammatory markers, whereas linear regression analysis was used to further explore their relationship, which should be checked in advance for multicollinearity among the variables. A *p*-value of < 0.05 was considered to indicate statistical significance.

Post-hoc power analysis for primary outcomes was performed with GPower (*α* = 0.05).

## Results

In total, 120 study participants were enrolled, including 40 healthy individuals (healthy group), 40 patients with type 2 diabetes without diabetic retinopathy (NDR group), and 40 patients with type 2 diabetes with macular edema (DME group). The mean ages of the three groups were 55.97 ± 11.43, 56.00 ± 11.13, and 55.92 ± 11.45 years, respectively. No significant differences were observed with regard to age, sex, or presence of hypertension among the three groups (*p* = 1.000, *p* = 1.000, *p* = 0.490, respectively). The mean BMI values of the healthy, NDR, and DME groups were 23.60 ± 2.46, 25.21 ± 3.59, and 24.92 ± 2.84 kg/m^2^, respectively, and the mean HbA1c was 5.56 ± 0.30%, 8.85 ± 1.95%, and 9.38 ± 2.09%, respectively, with significant differences among the three groups (*p* = 0.042 and *p* < 0.0001, respectively) (Table 1).

**Table 1:** Comparison of baseline data among the three groups.

Variable	Healthy group (*n* = 40)	NDR group (*n* = 40)	DME group (*n* = 40)	*F/X^2^/Z*	*P*-value
Age, years, mean ± SD	55.97 ± 11.43	56.00 ± 11.13	55.92 ± 11.45	0.000	1.000
Sex, *n*%				0.000	1.000
Male	23 (57.5)	23 (57.5)	23 (57.5)		
Female	17 (42.5)	17 (42.5)	17 (42.5)		
High blood pressure, *n*%				1.425	0.490
No	29 (72.5)	24 (60.0)	27 (67.5)		
Yes	11 (27.5)	16 (40.0)	13 (32.5)		
BMI, kg·m^−2^, mean±SD	23.60 ± 2.46	25.21 ± 3.59	24.92 ± 2.84	3.262	0.042
HbA1c,%, mean±SD	5.56 ± 0.30	8.85 ± 1.95	9.38 ± 2.09	117.120	<0.0001
NLR, median (IQR)	1.46 (1.18 ~ 1.99)	1.68 (1.34 ~ 2.53)	2.36 (1.82 ~ 3.16)		<0.0001
				1.905	P^*^ = 0.057
				4.970	P^**^<0.0001
				2.810	P^***^ = 0.005
PLR, median (IQR)	95.71 (85.30 ~ 133.56)	103.02 (83.44 ~ 122.07)	125.62 (94.36 ~ 150.78)		0.025
				0.183	P^*^ = 0.855
				2.242	P^**^ = 0.025
				2.439	P^***^ = 0.015
MLR, median (IQR)	0.17 (0.12 ~ 0.25)	0.22 (0.19 ~ 0.31)	0.21 (0.15 ~ 0.27)		0.002
				3.484	P^*^<0.0001
				1.906	P^**^ = 0.057
				1.828	P^***^ = 0.068
SII, median (IQR)	295.54 (229.02 ~ 397.50)	319.49 (244.53 ~ 481.31)	538.21 (389.76 ~ 732.96)		<0.0001
				0.953	P^*^ = 0.341
				4.869	P^**^<0.0001
				3.926	P^***^<0.0001

NDR, type 2 diabetes without diabetic retinopathy; DME, type 2 diabetes with macular edema; SD, standard deviation; BMI, body mass index; HbA1c, hemoglobin A1C; NLR, neutrophil-to-lymphocyte ratio; PLR, platelet-to-lymphocyte ratio; MLR, monocyte-to-lymphocyte count ratio; SII, systemic immune-inflammation index; *, healthy group compared with NDR. **, healthy group compared with the DME group; ***, NDR group compared with the DME group.

The NLR values of the DME group were significantly different from those of the healthy and NDR groups (*p* < 0.0001 and *p* = 0.005, respectively). Similarly, PLR values in the DME group were significantly different from those in the healthy and NDR groups (*p* = 0.025 and *p* = 0.015, respectively). MLR values were significantly different between the healthy and NDR groups (*p* < 0.0001). For SII values, significant differences were observed between the DME group and both the healthy and NDR groups (*p* < 0.0001 and *p* < 0.0001, respectively; Table 1).

When comparing the BCVA, CMT, NLR, PLR, MLR, and SII of patients in the DME group before the first anti-VEGF treatment and after the third anti-VEGF treatment, significant differences were observed in BCVA and CMT (*p* < 0.0001 and *p* < 0.0001, respectively). The BCVA was significantly improved after treatment, whereas the CMT was significantly reduced. There were no significant differences in NLR, PLR, and SII before and after anti-VEGF treatment (*p* = 0.882, *p* = 0.778, *p* = 0.510, respectively), but a significant difference existed in MLR before and after treatment (*p* = 0.003) (Table 2).

**Table 2:** Comparison of BCVA, CMT, and inflammatory markers before and after anti-VEGF treatment in the DME group.

Variable	Pre-treatment (*n* = 40)	Post-treatment (*n* = 40)	*Z*	*P*-value
BCVA (logMAR)	0.96 (0.60 ~ 1.12)	0.70 (0.52 ~ 1.00)	4.206	<0.0001
CMT, μm	404.00 (317.50 ~ 519.00)	271.50 (245.50 ~ 319.75)	5.511	<0.0001
NLR	2.36 (1.82 ~ 3.16)	2.37 (1.78 ~ 3.09)	0.148	0.882
PLR	125.62 (94.36 ~ 150.78)	126.85 (90.61 ~ 154.47)	0.282	0.778
MLR	0.21 (0.15 ~ 0.27)	0.24 (0.18 ~ 0.31)	3.011	0.003
SII	538.21 (389.76 ~ 732.96)	508.15 (357.38 ~ 678.37)	0.659	0.510

BCVA, best-corrected visual acuity; CMT, central macular thickness; NLR, neutrophil-to-lymphocyte ratio; PLR, platelet-to-lymphocyte ratio; MLR, monocyte-to-lymphocyte count ratio; SII, systemic immune-inflammation index.

**Figure 2:**
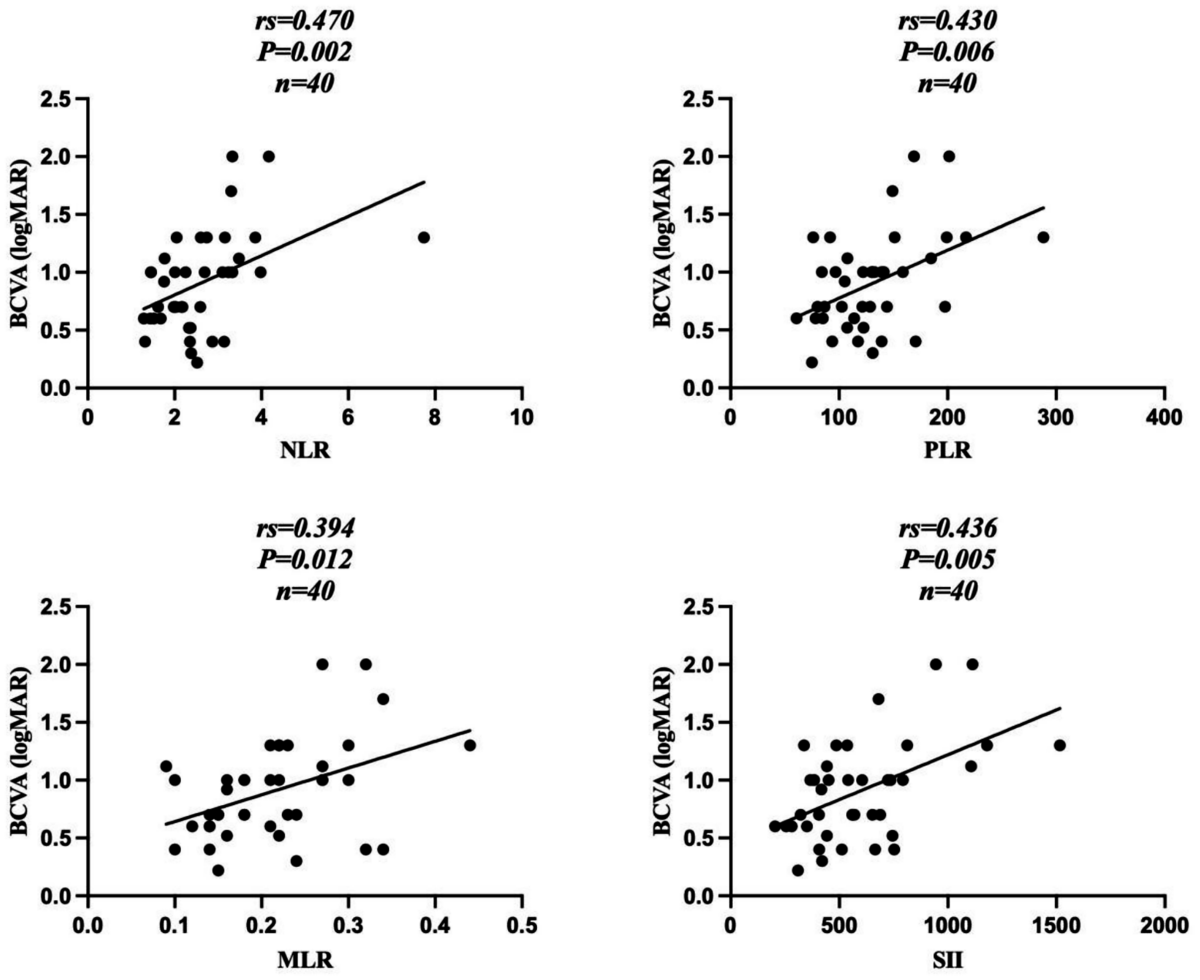
Correlation between BCVA and leucocyte ratios in the DME group before anti-VEGF treatment. DME, type 2 diabetes mellitus complicated with macular edema; BCVA, best-corrected visual acuity; NLR, neutrophil-to-lymphocyte ratio; PLR, platelet-to-lymphocyte ratio; MLR, monocyte-to-lymphocyte count ratio; SII, systemic immune-inflammation index.

**Figure 3:**
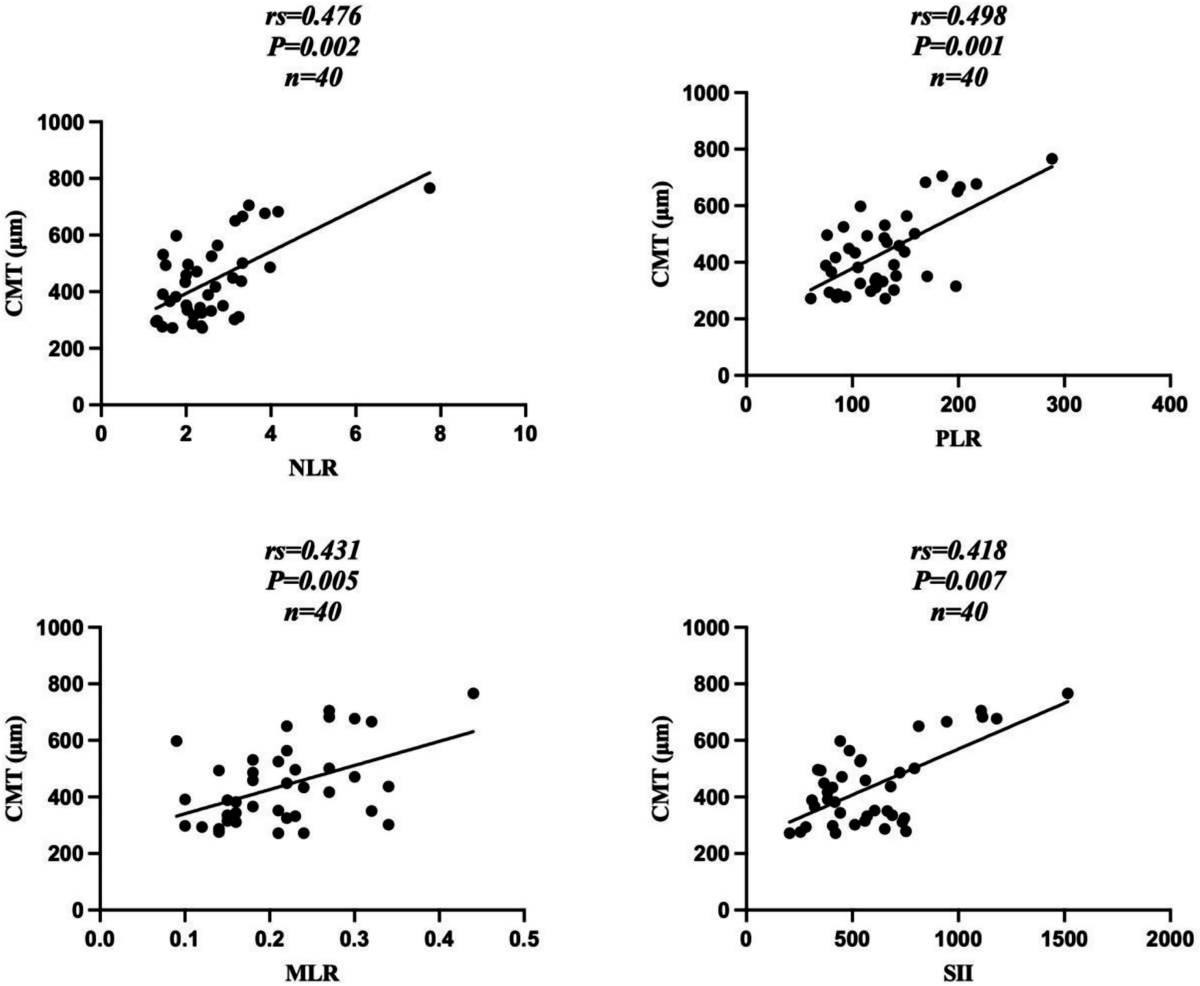
Correlation between CMT and leucocyte ratios in the DME group before anti-VEGF treatment. DME, type 2 diabetes mellitus complicated with macular edema; CMT, central macular thickness; NLR, neutrophil-to-lymphocyte ratio; PLR, platelet-to-lymphocyte ratio; MLR, monocyte-to-lymphocyte count ratio; SII, systemic immune-inflammation index.

Correlation analysis revealed that BCVA in the DME group before anti-VEGF treatment was positively correlated with NLR (*r*_s_ = 0.470, *p* = 0.002), PLR (*r* = 0.430, *p* = 0.006), MLR (*r* = 0.394, *p* = 0.012), and SII (*r* = 0.436, *p* = 0.005) (Figure 2). CMT before anti-VEGF treatment was positively correlated with NLR (*r*_s_ = 0.476, *p* = 0.002), PLR (*r* = 0.498, *p* = 0.001), MLR (*r* = 0.431, *p* = 0.005), and SII (*r* = 0.418, *p* = 0.007) (Figure 3).

To further analyze the relationship between BCVA and CMT and NLR, PLR, MLR, and SII in the DME group, linear regression analysis was performed between BCVA and CMT after anti-VEGF treatment and NLR, PLR, MLR, and SII before treatment. Univariate linear regression analysis showed that NLR, PLR, MLR, and SII were significantly correlated with BCVA (*p* = 0.003, *p* = 0.007, *p* < 0.0001, and *p* = 0.011, respectively) and CMT (*p* < 0.0001, *p* = 0.001, *p* = 0.001, and *p* = 0.004, respectively). In other words, larger NLR, PLR, MLR, and SII values corresponded to worse BCVA and thicker CMT after DME treatment. In multivariate linear regression analysis, only NLR was significantly associated with CMT (*p* = 0.001) (Table 3).

**Table 3:** Risk factors affecting BCVA and CMT after anti-VEGF therapy in the DME group.

Variable	Univariate linear regression (*n* = 40)			Multivariate linear regression (*n* = 40)			Univariate linear regression (*n* = 40)			Multivariate linear regression (*n* = 40)		
	B (BCVA)	95%CI (BCVA)	*P*-value (BCVA)	B (BCVA)	95%CI (BCVA)	*P*-value (BCVA)	B (CMT)	95%CI (CMT)	*P*-value (CMT)	B (CMT)	95%CI (CMT)	*P*-value (CMT)
NLR	0.107	0.039 ~ 0.175	0.003	0.114	−0.034 ~ 0.262	0.127	52.614	32.801 ~ 72.427	<0.0001	72.683	30.715 ~ 114.651	0.001
PLR	0.002	0.001 ~ 0.004	0.007	0.001	−0.002 ~ 0.005	0.347	0.956	0.396 ~ 1.515	0.001	0.507	−0.366 ~ 1.380	0.247
MLR	0.996	−0.074 ~ 2.065	<0.0001	−0.528	−2.089 ~ 1.034	0.497	573.172	238.329 ~ 908.016	0.001	−65.443	−505.104 ~ 377.218	0.766
SII	0.000	0.000 ~ 0.001	0.011	0.000	−0.001 ~ 0.001	0.711	0.144	0.049 ~ 0.240	0.004	−0.156	−0.330 ~ 0.018	0.077

DME, type 2 diabetes with macular edema; BCVA, best-corrected visual acuity; CMT, central macular thickness; NLR, neutrophil-to-lymphocyte ratio; PLR, platelet-to-lymphocyte ratio; MLR, monocyte-to-lymphocyte count ratio; SII, systemic immune-inflammation index.

To assess the statistical power, a *post-hoc* analysis was carried out in GPower for the primary outcomes (NLR, PLR, MLR, and SII) at *α* = 0.05 (Table 4).

**Table 4:** Post-hoc power analyzes in this study.

Variable	Total sample size	Cohen’s f	Statistical power (1−*β*)
NLR	120	0.418	98.0%
PLR	120	0.420	98.0%
MLR	120	0.367	93.0%
SII	120	0.483	99.6%

NLR, neutrophil-to-lymphocyte ratio; PLR, platelet-to-lymphocyte ratio; MLR, monocyte-to-lymphocyte count ratio; SII, systemic immune-inflammation index.

## Discussion

In this study, we evaluated the predictive value of peripheral white blood cell counts and ratios for the efficacy of anti-VEGF treatment (ranibizumab) in patients with DME. We found that in patients with DME, the proportion of peripheral blood leukocytes and the systemic immune-inflammation index (SII) increased after three anti-VEGF injections and were positively correlated with both BCVA and CMT. These results suggest that serum inflammatory markers may be considered biomarkers of successful DME treatment and can be used to predict the effectiveness of intravitreal anti-VEGF in patients with DME before treatment. A particularly critical finding is that an elevated NLR emerged as a powerful and independent predictor of BCVA and CMT after anti-VEGF therapy in the DME group, after adjusting for key clinical covariates. This indicates that NLR provides unique prognostic information beyond standard parameters. The study was conducted at a single center and focused on a Chinese population. Therefore, the generalizability of the findings to other ethnic groups and clinical settings may need further validation. *Post-hoc* power analysis of the primary outcomes demonstrated robust statistical power (> 0.80). This indicates that the study was adequately powered to detect the observed effect size, which increases confidence in the reliability of the results.

The pathogenesis of DME is extremely complex and has not yet been fully elucidated. Currently, it is believed that DME begins with retinal hypoxia, which induces the upregulation of VEGF expression, resulting in increased retinal capillary permeability. VEGF plays a key role in the pathogenesis of DME (21). Since its approval by the FDA for intravitreal administration in 2011, ranibizumab has been widely used to treat DME and is currently considered the first-line treatment option for DME (22). In our study, we used BCVA and CRT as measurements to evaluate anti-VEGF treatment efficacy and found that patients with DME showed significant improvement in both BCVA and CRT after anti-VEGF therapy. However, some patients did not respond to this treatment and might show worsening of the disease (23). This suggests that other factors besides VEGF may be involved in the pathogenesis of DME. Inflammation plays an important role in the formation of DME (24). DME is associated with certain serum inflammatory factors, but the exact mechanism of inflammation at the local level remains controversial. As markers of systemic inflammation, NLR, PLR, MLR, and SII have been used as prognostic indicators for various chronic diseases. Peripheral blood leukocytes can affect the occurrence and progression of DR (25). Elevated peripheral blood neutrophil counts are associated with the presence and severity of DR. Neutrophil-mediated inflammation may play a key role in the pathogenesis of DR (26). Previous studies have confirmed the presence of elevated NLR in the peripheral blood of patients with DR (27, 28). Moreover, higher NLR levels before anti-VEGF treatment were associated with poor BCVA after treatment (29). Unfortunately, only one indicator, NLR, was used as a biomarker in this study. SII is thought to be associated with cardiovascular disease (30), cancer (31), autoimmune diseases (32), and metabolic diseases (33). The relationship between platelets and inflammation is also receiving increased attention (34). Zhou et al. (35) found that the number of retinal hyperreflective foci on OCT correlated with SII, NLR, and PLR. This supports the theory that inflammation plays a significant role in the pathogenesis of DME. Chen et al. (36) also found that the systemic inflammation indices NLR, PLR, MLR, SII, and C-reactive protein were significantly different in patients with DME at different periods. These previous findings were consistent with our results. In our study, we further confirmed the correlation between NLR, PLR, MLR, SII, and the effect of anti-VEGF treatment, and used a regression analysis model to predict the effect of the treatment.

Interestingly, except for MLR, statistical differences were not found in other inflammatory indicators before and after treatment, which may be because the small dose of local vitreous cavity drug therapy is not enough to affect systemic changes in peripheral blood. While our data did not show a correlation between changes in inflammatory indices and treatment response, the baseline elevation of NLR, PLR, MLR, and SII in untreated DME patients underscores the role of systemic inflammation in the disease’s pathogenesis. This can be explained by several interconnected mechanisms. The neutrophils and monocytes represented in these indices are potent producers of VEGF and pro-inflammatory cytokines (e.g., IL-1β and TNF-*α*), which disrupt tight junctions and increase vascular permeability in the retina. Furthermore, platelets contribute to this process by releasing similar mediators upon activation. The SII, as a composite marker, effectively captures the interplay between these pathways. Our findings support the hypothesis that DME exists within a state of chronic low-grade systemic inflammation, which may prime the retinal vasculature for leakage. The lack of correlation with anti-VEGF response suggests that while this inflammatory background is a key permissive factor for DME development, the acute suppression of VEGF alone may be sufficient to resolve edema regardless of the systemic inflammatory burden, or that our study was underpowered to detect such a relationship. Future studies are needed to explore whether these indices predict responses to therapies targeting broader inflammatory pathways.

The effect of anti-VEGF therapy directly affects the treatment compliance of DME patients (37). Due to the high price of anti-VEGF drugs, patients tend to interrupt treatment if their visual acuity is not significantly improved after receiving one treatment, which may lead to irreversible damage to their visual acuity. Beyond their pathophysiological significance, the systemic inflammatory indices (NLR, PLR, MLR, and SII) investigated in this study offer considerable advantages in potential translation into real-world clinical practice. Their primary feasibility lies in the fact that they are derived from a routine, inexpensive, and universally available complete blood count (CBC) test. This makes them vastly more accessible and cost-effective than specialized retinal imaging modalities such as OCT angiography or more complex, proprietary molecular biomarkers such as aqueous humor cytokine levels (e.g., IL-6 and VEGF) or genetic markers. Unlike these latter options, which require specialized equipment, invasive procedures, or advanced laboratory techniques, NLR, PLR, MLR, and SII can be calculated from existing data in almost any clinical setting, including primary care and underserved areas, facilitating rapid risk stratification and referral. In conclusion, while these circulating indices may not replace the precision of gold-standard ocular imaging or the mechanistic insight of intraocular biomarkers, they may serve as excellent, low-cost, and accessible screening and stratification tools. They could help identify DME patients with a significant systemic inflammatory burden, who might benefit from more frequent monitoring or adjunctive anti-inflammatory therapies, thereby paving the way for more personalized and efficient management in diverse healthcare settings.

The study had several limitations. First, the sample size was not predetermined, and the findings should be interpreted as preliminary, warranting confirmation in larger, prospectively designed studies. Second, blood tests are susceptible to unknown confounding factors, which may also affect the results. Finally, peripheral blood cells are of limited use in predicting treatment outcomes in DME patients, and more evidence is needed to further support this.

## Conclusion

Our findings further elucidated the pathogenesis of DME. Our study found that systemic inflammatory indices (NLR, PLR, MLR, and SII) were elevated in DME patients. While anti-VEGF therapy led to the anticipated anatomical and functional improvements (reduced CMT and improved BCVA), the magnitude of change in these inflammatory indices did not demonstrate a significant association with the treatment response. This suggests that the systemic inflammatory state reflected in these indices may not be a primary driver or a predictive biomarker for the short-term efficacy of anti-VEGF therapy in DME. However, only NLR emerged as an independent predictor in the multivariate analysis. The treatment of DME should be individualized after fully determining its complex mechanism. The study of inflammatory cells and factors may provide a new pathway for the management of DME. Furthermore, the research results based on the 3-month follow-up are related to short-term outcomes, and their applicability to long-term prognosis remains to be determined.

## Data Availability

The raw data supporting the conclusions of this article will be made available by the authors, without undue reservation.
